# Intensity Dependent Confidence Intervals on Microarray Measurements of Differentially Expressed Genes: A Case Study of the Effect of MK5, FKRP and TAF4 on the Transcriptome

**Published:** 2007-07-17

**Authors:** Werner Van Belle, Nancy Gerits, Kirsti Jakobsen, Vigdis Brox, Marijke Van Ghelue, Ugo Moens

**Affiliations:** 1 Department of Microbiology and Virology, Section Virology, Faculty of Medicine, University of Tromsø, Norway; 2 Department for Medical Genetics, University Hospital Tromsø, Northern Norway

**Keywords:** Microarray analysis, confidence intervals, measurement errors, gene-array, upregulation, downregulation, differential expression

## Abstract

To perform a quantitative analysis with gene-arrays, one must take into account inaccuracies (experimental variations, biological variations and other measurement errors) which are seldom known. In this paper we investigated amplification and noise propagation related errors by measuring intensity dependent variations. Based on a set of control samples, we create confidence intervals for up and down regulations. We validated our method through a qPCR experiment and compared it to standard analysis methods (including loess normalization and filtering methods based on genetic variability). The results reveal that amplification related errors are a major concern.

## 1 Introduction

The transcriptome contains all the mRNA transcripts in a specific cell(type) under certain conditions. Depending on these conditions, the amount of individual mRNA may vary. Microarray studies allow the rapid identification of many transcripts in cells under controlled conditions and can be used to compare expression patterns of genes between cell systems under different circumstances. For example, one can monitor the transcripts in normal versus diseased cells, or control cells versus cells lacking a specific gene or overexpression of a particular protein or a mutated form of a protein.

Analysis of such differential expression experiments often involves normalization (Smyth and Speed, 2003; Cleveland, Grosse and Shyu, 1992), data filtering (Dudoit, Yang, Callow et al. 2000) and reporting measured changes. Subsequently, neural networks (Sawa and Ohno-Machado, 2003), eigenvalue decomposition (Sanguinetti, Milo, Rattray et al. 2005; Alter, Brown and Botstein, 2000) and various cluster algorithms (Bilu and Linial, 2002; Nakaya et al. 2001) can help to elucidate the results. Annotation of genes with their cellular location, function or gene-category/sequence then provides more insight into the effects of the altered gene expression.

In this paper we focus on the measurement processes involved in such experiments. Microarrays contain a number of error-sources (Ramdas, Coombes, Baggerly et al. 2001), some of them physical (quenching (Kubista, 1994; Randolph and Waggoner, 1997)), some chemical (hybridization), some related to the electronics (gating (Schäferling and Nagl, 2006), dynamic range (de la Nava, van Hijum and Trelles, 2006), saturation (Lyng, Badiee, Svendsrud et al. 2004)). In most microarray experiments the measurement errors remain unknown, but they are widely believed to follow Lorentz distributions (Press, Teukolsky, Vetterling et al. 2003; Brody, Williams, Wod et al. 2002).

The general assumption with such experiments is that ’strong signals are better signals’. However, given the realization that cell systems might propagate noise throughout genetic pathways, we hypothized that strong signals might be subject to greater measurement errors. Instead of having an absolute error one would then find a relative error as well. To study such errors we conducted a number of experiments that all included a control sample. That control sample would simultaneously account for experimental-, biological- and machine-related variations, after which we could assess the error distributions on an intensity specific basis. Based on the error model, our technique reports confidence intervals for up/down regulation.

This study is set in the context of three experiments. The first involves the mitogen-activated protein kinase-activated protein kinase-5 (MAPKAPK5 or MK5). This this protein kinase belongs to the MAPK signaling pathway and at present, knowledge of its role in cellular processes remains limited (Gaestel, 2006). To examine a possible effect of MK5 on transcription, we constructed a doxycycline-inducible PC12 cell line that allowed inducible expression of a constitutive active form of MK5 (MK5*_L_*_337_*_A_*). RNA was purified from three independent samples of cells grown in the presence of doxycycline (no expression of activated MK5) and from three independent samples of cells in which the expression of MK5 was turned on by removal of doxycycline. Each microarray slide (KTH Rat 27k Oligo Microarray-Operon ver3.0) was loaded with one sample uninduced (Cy5) and one sample induced (Cy3) (for a reference on Cy5/Cy3 see (Mujumdar, Ernst, Mujumdar et al. 1993)). We added a fourth slide containing two induced samples as a control for measurement errors.

The second experiment involves the TATA binding protein Associated Factor 4 (TAF4). The transcription factor TFIID is a multiprotein complex composed of the TATA box-binding protein (TBP) and multiple TBP-associated factors (TAFs). TFIID plays an essential role in mediating transcriptional activation by gene-specific activators. TAFs have been postulated to exert several important roles in transcription acting as core promotor specificity factors and co-activators. Genetic studies in vertebrate cells also point to an essential role of TAFs in cell cycle progression (Thomas and Chiang, 2006; Naar, BD and Tjian, 2001; Albright and Tjian, 2000; Davidson, Kobi, Fadloun et al. 2005). Using siRNAs we measured the influence of TAF4 depletion on the transcriptome^1^. These experiments were performed in HeLa cells and SK-N-DZ cells. For each cell type we used 4 slides with scrambled siRNAs and 4 slides with TAF4-directed siRNA. The microarrays relied on DIG (digoxigenin) labeling.

The third experiment focuses on a putative glycosyltransferase. A number of congenital muscular dystrophies (CMD) are now known to be associated with mutations in genes encoding for proteins that are either putative or determined glycosyltransferases. This supporst the idea that aberrant posttranslational modifications of proteins may represent a new mechanism of pathogenesis in the muscular dystrophies. One of these genes, fukutin-related protein (FKRP), is thought to be coding for a putative glycosyltransferase, but its function has not yet been established (Brockington, Blake, Brown et al. 2002). To evaluate the possible effect of FKRP on transcription we transfected C2C12 cells with siRNA that targets FKRP. The results of the transfection were measured using microarray analysis using DIG labeling. Table 1 gives an overview of the different experiments.

## 2 Analysis Method

The presented analysis method measures the variance of a control sample, then uses it to model an intensity dependent error distribution and based on that, defines confidence intervals for each individual spot, or group of spots. Regulations are reported as terms within a confidence interval of 95%. Conversion to ratios can be performed as necessary.

### Acquiring the error model

To acquire the error model, one can employ two techniques. The first supplies a number of identical pairs of biological samples and puts them on different slides. For instance, one slide can contain the TAF4 downregulated transcript, while another slide contains the normal transcript. One can then use the inter-slide variance to develop an error model. A second approach, and the one used for the MK5 experiment, acquires the error on the regulation difference. In this setup, one provides the same sample for red and green. Because red and green have the same content, one expects both channels to be equal for all spots. In the discussion below we assume that red and green name two samples that ought to be compared. Whether they are using Cy5/Cy3 staining or DIG labeling is irrelevant for the discussion.

**Figure 1:**
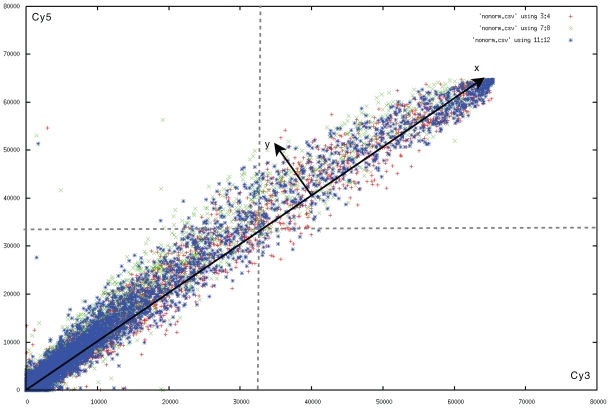
Scatterplot of the control slides and the two measurements of the MK5 experiments. The red points are from slide 1. The green points are from slide 3. The blue points are from the control slide. Horizontally the red channel is set out, vertically the green. The bend is due to quenching (Kubista, 1994). The variance of the control slide can be observed in the width of the blue area. It increases up to 32768 (indicated with gray dotted lines), after which it decreases again. In a perfect world, the control sample should have the same red as green value, and be a straight line.

Figure 1 plots the red and green channel of such a control slide. We find that the variance around the expected values increases together with the spot intensity. This phenomenon indicates relative errors, and is the main reason why one relies on a log-transform. However, in the second half (with red or green intensities larger than 32768) the variance decreases with increasing spots intensity. A partial reason for this might lie in the number of saturated pixels.

The above observation on the error distribution prohibits us to use a maximum likelihood estimation of the absolute and relative errors (Ideker, Thorsson, A.F.Siegel et al. 2000; Press, Teukolsky, Vetterling et al. 2003). Instead, we model a collection of error distributions: one for each intensity. A two-dimensional map will count the number of spots with a specific intensity and deviation. Spot intensity (set out horizontally) is calculated as the mean of the red and green channel. Spot deviation (set out vertically) is red subtracted from green. Afterwards, the algorithm normalizes the two-dimensional histogram so that each intensity has: a) a proper cumulative probability distribution and b) relies on enough samples to have a good estimate of the modeled error. This process is detailed in section 7 and results in two functions *F* and *G*. They produce respectively a probability distribution and cumulative probability distribution for each intensity (*x*).

$$G(x)(y)=P(r-g<y\;\text{with}\frac{r+g}{2}=x)$$

**Figure 2:**
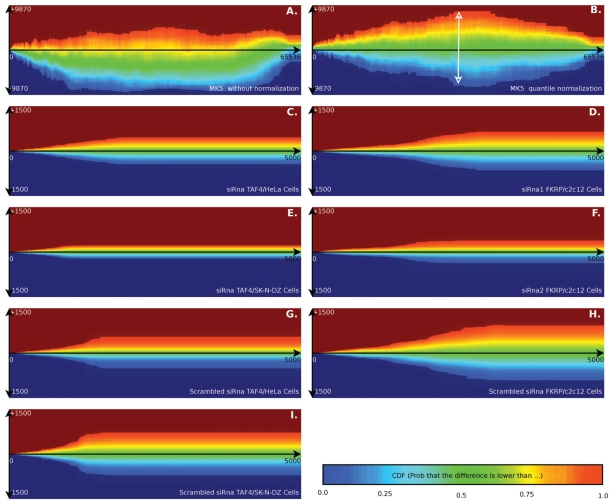
Error Distribution of various up/down regulation experiments. Horizontally the spot intensity is set out. Vertically the measurement error is set out as a cumulative distribution function. The cumulative distribution expresses the probability that a specific difference will occur due to experimental, biological or measurement variations. The colors are more intense within the 95% confidence interval. With such a diagram one can to determine the limits in which a regulation is very likely to fall. The multiple diagrams are measurement errors obtained from different experiments and different machines. The MK5 sample was Cy5/Cy3 stained and scanned on a Tecan scanner. All other samples were DIG labeled and scanned on an Applied Biosystems 1700 microarray scanner. As an example how to read the diagrams: in the MK5 diagram (top right) we find that the biological variation is larger for spots with intensity 32768. If a measured spot has intensity 32768, then its 95% confidence interval on the difference between the two channels is around[−9000, 9000] (marked with a white arrow).

For illustrative purposes, we added *x* and *y* labels to Figure 1. Figure 2 plots the error distribution of the MK5 experiment. When the error model is obtained from different slides then the probability distribution *F* (and associated cumulative distribution *G*) is based on the error model of each slide and convolved accordingly.

### Confidence intervals on one measurement

Assuming that the probability distribution *f* expresses the error distribution of a specific spot, and that *r* is the real (but unknown) regulation, then our measurement *m* will report a value in the range *m* = *r* + ε, in which ε satisfies *f*. In other words, instead of measuring the real regulation, we will always measure the real regulation with some extra unknown error. Since we know *m* and have some understanding of ε (its distribution) we can state that *r* = *m* − ε. Thus, by determining a confidence interval on ε we can report a confidence interval on *r* as well.

A 95% confidence interval for spots with intensity *x* is given as [*G*^−1^ (*x*) (0.025) : *G*^−1^ (*x*) (0.975)]. If a spot measures as *m*, then in 95% of the cases, the real regulation falls within

$$\left[m-G^{-1}(|m|)(0.025):m-G^{-1}(|m|)(0.975)\right]$$

### Reporting regulations

A widely accepted method for quantitative measurement are log-ratios. Despite widely used, they have a number of important limitations. First, the log ratio cannot capture information such as the measurement error. For instance the ratio 2/1 has probably more errors involved than 2000/1000. The log_10_ ratio will report 0.3 regardless. Secondly, the log ratio has numerical problems near zero. An up- or down-regulation from zero to 1416 might make biological sense but it seems inappropriate to express it as a (log-)ratio of ∞.

**Figure 3:**
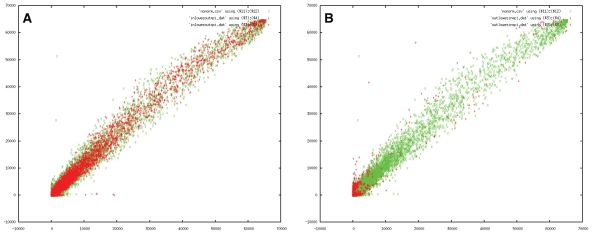
Plots illustrating the difference between standard filtered results (based on loess normalization and a consensus for both slides) and the filtering based on the confidence intervals for the MK5 experiment. **A**) the red spots are reported by the standard method but no longer by the confidence interval method. The green spots are the control slide, illustrating the large variance of the measurement. All spots omitted in the confidence interval method were too close to the measurement error to be useful. **B**) The red spots are those reported in the confidence interval method but not in the standard analysis. The green spots again represent the control slide.

To approach these challenges, our method reports the measured regulation as the difference between two slides, thereby including the lowest and highest expected differences (Table 2). In many cases this leads to an up- *or* downregulation. Such non-sensical regulations ought to be filtered out since the possible error outweighs the actual measurement. E.g. a confidence interval of [−1950 : 1950] for a spot with a regulation of −500 indicates that the real regulation-difference will range within [−2450 : 1450]. Figure 3A illustrates a set of points omitted due to such filtering.

When a consensus on the regulation exists (lowest boundary and highest boundary have the same sign), we can calculate the regulation ratios by assuming that either red or green could have been fully responsible for the measurement error. In such extreme cases the highest ratio can have a value of ∞.

### Confidence intervals on multiple measurements

When multiple measurements are available, we can make the final confidence intervals smaller by convolving their respective probability functions. Section 7 covers the details. Table 2 illustrates the combination of oligosequences belonging to the same gene and consequently reports smaller confidence intervals.

As an illustrative example of the advantage of combining the different probability distributions we investigate gene #34 (Table 2). The microarray measures this gene using two distinct probes, labeled Rn30006190 and Rn30021393. On slide 1, Rn30006190 has an upregulation in the range [−455 : 2504] (measured as 999). On slide 2, it has an upregulation in the range [−256, 675] (measured as 184). On slide 1, Rn30021393 has an upregulation in the range [−815 : 3106] (measured as 1017). On slide 2, it has an upregulation in the range [−1080 : 4131] (measured as 1623). None of these individual measurements can tell us something about the gene regulation since they all could have been downregulated as well. However, by combining their error distributions we are able to report that the overall gene is upregulated with at least a 6% increase and at most a 4.6 times increase (last row of Table 2).

## 3 Validation

We validated our method by means of qPCR and by comparing it to standard analysis protocols. For MK5 this analysis was performed at the Microarray facility in Tromsø. For the FKRP and TAF4 experiments, this analysis was performed by UNIGEN (Trondheim).

### Quantitative PCR

To validate the regulations we found in the TAF4 experiment, we selected 22 genes and monitored their transcript levels by quantitative PCR (qPCR). Such qPCR results should be treated with caution. First, it is an inherent different measurement technique and thus it is unexpected that the results will completely fall within the reported confidence intervals. Secondly, the quantitative PCR experiment is often based on a new batch of cells, which means that the transfection efficiency can be different, and thus the actual results as produced in the qPCR can be a ratio higher or lower. A new batch was used for the TAF4 HeLa cells. The SK-N-DZ cells were based on the same batch. To account for the transfection efficiency, we performed a least square fit of the qPCR results to the microarray results. Thirdly, the primer sequences can be slightly different leading to different measurement efficiencies. Fourth, the housekeeping gene used in the qPCR experiment can be indirectly linked with the genes we measure, leading to a gene specific bias. And as a last remark, since we do not have an error model of the qPCR measurements, the dynamic range of the housekeeping gene might put a limitation on the qPCR accuracy. Notwithstanding these considerations, we performed 22 qPCR experiments, which confirmed that our technique is a valuable analysis method. Table 4 summarizes the results.

From the 22 measurements, 3 were not used because we could doubt both the PCR and microarray results. In particular, a number of qPCR measurements could be considered up or down-regulated depending on the analysis process followed (e.g. mean of ratios versus ratio of means). From the 19 remaining genes, 12 were fully correct, that is, the qPCR results fell within the reported confidence interval. For 2 genes, the predicted upperbound was too low. For 3 genes, the microarray reported strong regulations, however the qPCR measurement was unable to measure the exact value because the CP values were too large. For these genes it is very likely that the microarray reported correct. One gene did not match between both experiments. And for 1 gene the microarray experiments reported a confidence interval that was substantially larger than the qPCR value.

In the strictest sense (upperbounds and lower-bounds match), our method was able to match 79% of the qPCR results. If one is satisfied with proper lower bounds, then 89% of the results were reported accurately.

### FKRP and TAF4

Next to the qPCR validation, we compared our method to a blind analysis by other groups. The blind analysis for the FKRP and TAF4 experiments followed the guidelines of Allison, Cui, Page et al. 2006. The PCA analysis revealed no outlier for any of the slides. The analysts attempted to gage the genetic variations (abbreviated: genvar) between the different slides and then report those that changed significantly. For the TAF4 HeLa cells experiment, the genvar error model reduced the dataset to 70 significant genes, while the intensity dependent analysis (abbreviated: indep) retained 2497 genes^2^. Five genes were only reported in the genvar set. Those 5 were all below the average gene intensity and the mismatch may be due to the normalization differences (quantile vs Applied Biosystems) or microarray outliers. We would liked to have validated those 5 mismatches through qPCR, but no probe sequences, nor gene annotations were available, so we could not verify them. The previous 22 qPCR measurements did however include 3 genes that were reported in the genvar analysis. Two of these produced qPCR values with large CP values (thus with a high error rate), thereby offering little extra information. For the FKRP experiment there were no significant alterations which was, according to the report, due to the few samples we provided (4 replicas vs 3 replicas). The indep analysis reported 2977 regulations for the siRNA#1 group and 576 regulations for the siRNA#2 group.

Compared to a standard analysis, our method reported more genes. In the TAF4 experiment, we found 35× more genes than the standard analysis. Most of these genes could be validated with qPCR, leading to the conclusion that standard analysis methods may be too stringent.

### MK5

The standard microarray analysis, based on loess normalization (Cleveland, Grosse, and Shyu, 1992; Smyth and Speed, 2003), contained 27648 spots for each slide, of which 4007 pairs in agreement (both slides reporting the same qualitative regulation, being up or down). Based on both slides, our method only reported 1422 spots. Three hundred and eleven spots occurred in both methods, 1111 spots were unique to our analysis and 3696 spots were unique to the standard analysis.

To better understand the differences in reported genes, it is helpful to include a picture (Figure 3) that illustrates both the variance on the measurements and the samples we removed/retained.

The first consideration regards spots that occurs in the loess set but not in our analysis. Is there a good reason why we should not take those particular data points into account ? Figure 3A illustrates the spots that only occurred in the loess set (red) as well as the variance of the experiment (green). Clearly, the omitted spots were too close within the expected variance to be useful.

The second concern regards those spots that only occurred in our analysis. These are pictured in Figure 3B. The main reason why our method was more sensitive and could report them lies in the convolution of the error distributions of similar spots. This information was unavailable to the loess method since there we were forced to stick to a more rigid approach that both slides agreed qualitatively.

The last concern regards overlapping spots. All of them should report at least the same qualitative regulation. From the 311 spots, 10 failed to do so. Looking at the non-normalized data (Table 3) we find that all spots were correctly reported by the confidence interval method. The reason why the loess method failed, probably lies in the model fitting that will inevitable position certain spots at the wrong side of the zero-line (a ratio of 2 is after all closely located to zero when expressed as a log_10_ ratio).

## 4 Discussion

Our method was validated using qPCR and we found that it reports useful confidence intervals (79% correct, 89% when omitting the upper limit). We also found that the method surpasses standard methods in the number of genes it reports (×35 in our case).

### Difference between machines, cell lines and experiments

The sampling of the error distribution is specific to the gain of the acquisition hardware, the biological sample, the slide quality, slide manufacturer, supplier of the microarray hardware, temperature, sample handling and probably many more influences. Therefore, the error model must be developed for each specific experiment. This is illustrated in Figure 2, which visualizes the difference between a number of these variables.

1. We illustrated the technique on a knockdown of a gene as well as on a constitutive active gene. Figures 2A and B are the constitutive active MK5. Figures 2C, D, E and F are those with a knockdown of a gene. These figures also illustrate the technique on two different scanners. Figures 2A & B are made on a Tecan scanner with Cy5/Cy3 labeling. All others are made with DIG labeled slides scanned on an Applied Biosystems 1700 scanner.
2. Figures 2G, H & I versus Figures 2C, D, E and F illustrate the differences between scrambled siRNA and specific siRNA. The results show that scrambled siRNA introduces more variability in the cell system than previously anticipated. This might suggest that a scrambled siRNA alone as a negative control might not be sufficient, or will in a sense, reduce the number of useful results that can be obtained from this type of experiment.
3. We illustrated the technique on the same experiment, but with different cell types. Figures 2C, G are performed in HeLa cells, while Figure 2E, I plots the data from SK-N-DZ cells. Compared to the FKRP experiments, they reach their maximum variability point at lower intensities. Between the two different cell types we find that the SK-N-DZ cells reached their maximum variability point also at lower intensities.
4. Figure 2D plots siRNA#1 while Figure 2F plots the siRNA#2, which target slightly different FKRP mRNA. The small variations in Figure 2F might suggest that we would obtain more data from this experiment. This however is incorrect. For siRNA#2 we only obtained 576 valuable genes, while the siRNA#1 group produced 2977 genes. This probably happened due to either a bad transfection efficiency (leading to low variations, but also to little useful data) or a low siRNA#2 impact in general. This illustrates that the size of the error as such does not provide much information, it must always be related to the impact of the cell alteration itself.
5. Figures 2D, F, H are mouse survey gene arrays, while Figures 2C, E, G, I are human genome survey arrays. We find little overall impact of the type of array in the shape of the error plots.
6. Figure 2A is made using Cy5/Cy3 labeling without normalization. Figure 2B is the same figure but relying on quantile normalization. Figure 2C–I are based on the applied biosystem inter array normalization algorithm. The differences in confidence intervals between Figure 2A and Figure 2B illustrates how our algorithm can model the inter-filter effect (Kubista, 1994). Instead of having a flat ‘eye-shaped’ error model (Fig. 2B), one finds back a ‘banana-shaped’ error model. This means that the model is independent from a particular normalization to account for light reabsorption. Using confidence intervals, there is no particular need to perform separate dye specific normalizations.

Looking at these observations, we see that the machine fabricant and normalization algorithm have a major impact on the shape of the error plots. The type of cell perturbation, in our case, is a second major factor (scrambled siRNA vs specific siRNA). The specific cell lines (HeLa vs SK-N-DZ), actual genes (TAF4 vs FKRP) and type of microarray (mouse versus human) have a lesser impact on the overall shape of the error plot.

### Optimal areas of measurement

Looking at the results (Figure 2 and 3B), our observations do not support the general believe that ‘bright spots are good spots’. Actually, we find that intense spots are subject too much larger errors. Therefore we might wonder whether there are measurement areas that produce the most information. In our MK5 error model we find that the bright spots are the ones that should be removed from the data set since they are too close to the expected error, while the darker spots often fall outside the measurement error (see Fig. 3A). Figure 3B illustrates this further: contrary to what one would expect we find the largest collection of useful spots at the edges around the origin.

### Amplification errors seem to outweigh genetic variability

Given the considerations these days on genetic pathways and genetic variability, we now discuss how these two factors influence our analysis method. The first concern is that certain genes have a larger natural variability (unstable expressed genes) than other, more stably expressed, genes. Since our method does not assess genetic variability, it might omit significant changes in stably expressed genes if they are too close to each other. It might also report highly unstable expressed genes as significantly altered while, in reality, they might just have fallen outside the confidence interval by chance. While there may be such genes, our initial observations does not seem to be influenced by it. Our PCR results confirm our confidence intervals, which seems to indicate that the impact of genetic variability is much lower than anticipated. Instead we find that the experimental variability, cell perturbation and consequent amplification/propagation cascades outweighs natural genetic variability.

The second concern addresses genetic pathways: the gene expression pattern in a cell is the result of a cascade event, where products of primary gene transcripts can affect the expression of other genes. Of course, when measuring the same samples, one still expects to find the same values (e.g. in Figure 1, regardless of the gene linking, the control *should* be a straight line). However, if an error or a variability occurs in the initial perturbation, then it is not unexpected that this error will propagate along the same pathways. This effectively leads to a cascade of expression patterns, in which every step can reduce or increase the net output effect. In other words, the amount of transcribed gene can be dependent on the amount of transcripts of linked genes, but multiplied with an unknown factor. Very seldom will we find that one expression pattern produces a new expression pattern with exactly the same amount of transcripts. So, by pooling together a random set of transcripts based on their intensity, we substantially limit the impact of genetic pathways. In the worst case scenario, if there were a significant collection of dependent transcripts, all with the same expression levels, then they would be placed in the same intensity-slice, thereby sharpening the probability distribution on that slice. This would in turn lead to a list of genes that could contain non-significantly altered gene expressions. In our work, we did not find much evidence that our intensity-based pooling is inadequate and/or overly sensitive to genetic pathways. The entire collection of probability distributions was in all our experiments smooth without outliers.

### Lorentz distributions

We believe that the presented method makes a fair trade off between a full understanding of the gene linkages/variations (which is something we cannot measure with 3 or 4 slides) and error models that do not take such possibility into account at all. Standard microarray error models are often based on the log-scale of the two channels (red/green or slide1/slide2) (Brody, Williams, Wod et al. 2002; Huber, von Heydebreck and Vingron, 2004). The resulting distributions appear as a Lorentz distribution (Press, Teukolsky, Vetterling et al. 2003; Brody, Williams, Wod et al. 2002). However, such distributions cannot capture relative errors in the experimental process. This leads to standard error models that are too wide for low intensity spots and too small for high intensity spots.

## 5 Conclusion

We presented a method to analyze differences between groups of microarrays, such as often found in differential gene expression experiments. Instead of reporting one single number for each regulation, we report the regulation including its confidence interval. The confidence interval is obtained from an error model that must be measured within the experiment itself.

We compared our method to a standard analysis method and illustrated its capability to filter out spots that are too close to the error to be useful. For indicative purposes we compared the reported results to standard analysis methods. We also performed a limited qPCR experiment. Although a relative small number of samples have been investigated, they support the credibility of our analysis method.

## 6 Material and Methods

Manufacturers instructions are used unless stated otherwise.

### Constitutive active MK5 cell-line

To clone the cDNA sequence of MK5, we introduced two mutations in the pcDNA-HA-MK5*_WT_* plasmid (Seternes, Johansen, Hegge et al. 2002). Both used the Stratagene mutagenesis kit. The first mutation assured compatibility with the pTRE2 plasmid and used by using primer 5′-CCC-AAG-CTT-GAC-GCG-TCC-ATG-TAT-GAT-G-3′ and its complementary reversed primer. The second mutation turned the wt MK5 into a constitutive active MK5*_L_*_337_*_A_* mutant. The resulting MK5 cDNA sequences were excised by *MluI/NotI* digestion and cloned into the corresponding sites of pTRE2. We verified the plasmid by sequencing. Two 6-well plates with 5.10^5^ PC12 TetOff cells (BD Biosciences) were transfected with 14 μg of pTRE2-MK5*_L_*_337_*_A_* and 2 μg pTKHyg per well using lipo-fectamine 2000 (Invitrogen) (Pianese, Busino, Biase et al. 2002). After 3.5 h, the medium was changed and supplemented with 10 ng/ml Doxycycline (Sigma). 24 h after transfection, cells were transferred to 10 cm dishes with fresh medium and Doxycycline. 48 h after transfection, 100 μg/ml of Geneticin (Gibco) and 200 μg/ml Hygromycin B (Invitrogen) was supplied additionally to the medium. The cells were grown until visible colonies of resistant cells could be detected. From each plate two colonies were transferred in threefold dilution to a 96 well plate. For positive clones, we confirmed the transgene expression through reverse transcriptase-PCR and western blot. Cells were maintained in DMEM supplied with 10% horse serum and 5% fetal bovine serum, 2 mM L-glutamine, penicillin (110 units/ml) and streptomycin (100 μg/ml). Additionally, 50 μg/ml of Geneticin, 100 μg/ml Hygromycin B were supplied to maintain selection. To suppress HA-MK5*_L_*_337_*_A_* expression during ordinary cell culture, we added 10 ng/ml Doxycycline.

### TAF4/FKRP knock-down using siRNAs

SiRNAs introduced into the cells lead to degradation of mRNA having the complementary sequence, thereby silencing/depressing gene expression. SiRNAs were pre-designed and ordered from Qiagen (http://www.qiagen.com/). For the FKRP experiment, the siRNAs sequences targeted AACCTCCTAGTCTTCTTCTAT; AACCCAAAGACTGGAGCAACT. For the TAF4 experiments, the siRNA targeted AAGGCCTGTGGATACTCTTAA. Cells were plated at 10^5^ cells/ml into a 6-well dish. Because of different growth-rates, HeLa and C2C12 cells were transfected after 24 hours, while SK-N-DZ cells were transfected after more than 48 hours. Two different transfection mixes were made. Both included 90 vol% D-MEM(SBS). The first transfection mix contained 10 vol% TAF4 siRNA (30 nM siRNA/well). The second transfection mix contained 10 vol% scrambled siRNA. The different mixes were vortexed, 7.5 μl RNAiFect was added and then incubated for 15 minutes (room temperature). D-MEM was aspirated from the wells. Subsequently, 100 μl of the transfection mixture was added to each well in addition to 1.9 ml fresh D-MEM (10% FBS + antibiotics). We produced each transfection mix in triplicate. Twenty-four hours after transfection, RNA was to be extracted for further analysis. The same procedure was followed in the FKRP knockdown experiments.

### RNA extraction and cDNA synthesis

C2C12 (FKRP), HeLa (TAF4) and SK-N-DZ (TAF4) cells were plated at 2.10^5^ cells per well in a 6 well dish; MK5 stable cells at 5.10^5^cells per 6 well dish. For the TAF4 and FKRP experiment, cells were lysed by incubation in lysis buffer containing chaotropic salt and Proteinase K, after which RNA was isolated with the MagNA Pure Compact RNA system (Roche-Applied-Science). For the MK5 experiment, we used the Nucleospin II RNA isolation kit (Machery-Nigel). The Nanodrop ND-1000 (Nanodrop technologies Inc.) verified RNA concentrations and purity. One μg of RNA was reverse transcribed to cDNA using the iScript cDNA synthesis kit (Biorad) (MK5) and SuperScript^TMII^ from Invitrogen^TM^ (remaining experiments).

### Quantitative realtime PCR TAF4 related genes

We made 4 cDNA dilutions: 1:2, 1:5, 1:10 and 1:50. All were supplemented with mastermix, primers, probe and water. Relative expression for each target gene was normalized to GAPDH using the ^2^*^d^*CT method (Livak and Schmittgen, 2001). The expression differences between scrambled and normal siRNA were calculated by dividing the averages of each cell type. The qPCR experiments were performed on LightCycler 480 (Roche), with accompanying software version 1.2.0.0625.

### Microarray

The number of slides and their layout is provided in Table 1. For the MK5 experiment, we made 3 slides, each containing an induced (Cy3) and uninduced sample (Cy5). The 4th slide contained two induced samples. Samples were labeled with the 3DNA 350S HS labeling kit (Genisphere). Hybridized slides were scanned using the Genepix 4000B (Molecular Devices) with a constant gain of 950/800. We obtained more than 70% hybridization (measured as #spots > median + 1SD). Spots with too large an intensity (>90% of the maximum) or too large a regulation (> × 10) were removed. For standard analysis, we relied upon a blind analysis of the microarray facility in Tromsø, which used loess normalization (Cleveland et al. 1992). Our own analysis used quantile normalization (Dudoit et al. 2000). For the FKRP and TAF4 experiments, we used an Applied Biosystems 1700 scanner, with AB. v2.0 slides surveying respectively the mouse genome and human genome. UNIGEN in Trondheim performed a blind data analysis following the guidelines of (Allison, Cui, Page et al. 2006). This included quantile normalization on the raw machine output. Our analysis was based on the already normalized output of the Applied Biosystems scanner.

## 7 Detailed Analysis Method

### Notation

We denote every slide with a number which is placed top-right. The control slide is marked with a *c*. In the bottom-right we refer to either the red or green channel. Eg *d_r_^i^* refers to the red channel of spot *d* in slide *i*. Each channel must be measured, with or without quantile normalization, but always without taking the logarithm. The maximum measurable value is expressed as *C*, which typically is 65535 (this is the maximum value that can be expressed using 16 bits). The dataset is preferably already filtered for false positives. The norm of a spot *d* is written as

$$|d|:=\frac{d_{r}+d_{g}}{2}$$

The difference between the two channels is subscribed with a δ subscript. E.g. *d*_δ_ = *d_r_* − *d_g_*.

### Creating Histograms

We model the error distributions as a collection of histograms in function of spot intensity. We rely upon *s_x_* bins, each in which we store a histogram. We denote *h_x_* the histogram for bin *x*. It will cover all the spots within intensity range $\left[\frac{xC}{s_{x}},\frac{xC+C}{s_{x}}\right]$. The histogram *h_x_* counts the occurrences of a specific intensity. Using 2.*s_y_* bins, *h_x,y_* will cover all the spots for which the difference lies within $\left[\frac{yC}{s_{y}},\frac{yC+C}{s_{y}}\right]$. The creation of these histograms obviously starts with each *h_x,y_* = 0. The algorithm below calculates the 2 dimensional histogram.

$$\begin{array}{l} \text{foreach spot}d\\ x:=\frac{|d|s_{x}}{C}\\ y:=\frac{d_{\delta }s_{y}}{C}\\ h_{x,y}:=h_{x,y}+1 \end{array}$$

### Smoothing

After performing this process we smoothen out the distribution along the intensity axis. This ensures that each histogram contains a minimum amount of measurement-error measurements. The smoothing is performed adaptively by widening a window around each intensity until enough points are gathered. If we call *s_p_* the minimum mass of each histogram, then the algorithm below will create a smoothed collection of probability distributions and store it in *g*.

$$\begin{array}{l} \text{foreach intensity }X\\ w:=0\\ \text{do}\\ g\text{x}:=\sum_{x=X-w}^{X+w}h_{x}\\ w:=w+1\\ \text{while}\sum g\text{x}<s_{p}\\ g\text{x}:=\frac{g\text{x}}{\Sigma g\text{x}} \end{array}$$

In the above, the total mass of a histogram is written as ∑ *h*. The addition of histograms is the same as the addition of the counts in each bin. If *a* and *b* are two histograms then *c* = *a* + *b* ⇔ *c_i_* = *a_i_*+ *b_i_*. We use similar notation for division.

### Multiple measurements

Assume that we have a set of spots *M*, all measuring the same process (e.g. the same oligosequence, or the same gene), then we can define the overall measurement *m* as *m_r_* = ∑*_d_* _∈_ *_M_* *d_r_* and *m_g_* = ∑ *_d_*_∈ M_ *d_g_*. Then we also have that

$$m_{\delta }=\sum_{d\in M}d_{\delta }$$

The error distribution associated with a specific spot is written as *d̃*

For each value of we have an associated error *d*_δ_ distribution. The overall error distribution for *m_δ_* will consequently be the convolution of the underlying error distributions (written as ^*^).

$$\tilde{m}=\underset{d\in M}{*}\tilde{d}$$

### Confidence intervals

The confidence interval of κ associated with *m*, given the error distribution *m̃* is given by

$$\left[{\text{CDF}}_{\tilde{m}}^{-1}\left(\frac{1-\kappa }{2}\right),{\text{CDF}}_{\tilde{m}}^{-1}\left(\frac{\kappa +1}{2}\right)\right]$$

*m_l_* and *m_h_* are the lowest and highest boundaries for measurement *m*.

### Regulation Factors

Converting absolute regulation differences to regulation ratios requires that we assume that either *m_r_* or *m_g_* could have been fully responsible for the measurement error. This leads to the following possible regulation ratios:

$$\begin{array}{l} f_{1}=\text{if }m_{g}-m_{l}<0\;\text{then }\infty \;\text{else}\frac{m_{g}-m_{l}}{m_{g}}\\ f_{2}=\text{if }m_{g}-m_{h}<0\;\text{then }\infty \;\text{else}\frac{m_{g}-m_{h}}{m_{g}}\\ f_{3}=\text{if }m_{r}+m_{l}<0\;\text{then }\infty \;\text{else }m_{r}+m_{l}\\ f_{4}=\text{if }m_{r}+m_{h}<0\;\text{then }\infty \;\text{else }m_{r}+mh \end{array}$$

Min ({*f*_1_, *f*_2_, *f*_3_, *f*_4_}) reports the lowest possible regulation ratio. Max ({*f*_1_, *f*_2_, *f*_3_, *f*_4_}) reports the highest possible regulation ratio.

## Figures and Tables

**Table 1:** Overview of the different experiments.

Experiment	Constitutive Active MK5			FKRP Knockdown			TAF4 Knockdown			
**Labeling**	CY5/CY3			DIG			DIG			
**Microarray**	KTH Rat27K Oligo m.a, OperonV3.0 Tecan HS 4800 Genepix 4000B Genepix Pro 6.1.0.2			Applied Biosystems *Mouse* Genome Survey m.a.V2.0 DIG Labeling Applied Biosystems 1700 scanner			Applied Biosystems *Human* Genome Surveym. aV2.0 DIG Labeling Applied Biosystems 1700 scanner			
**Groups**	Normal (Cy5)	MK5 Induced (Cy3)	Control (Both)	siRNA #1	siRNA #2	Scrambled	SiRNA	Scrambled	siRNA	Scrambled
**Amount**	3	3	1	3	2	3	4	4	4	3
**Cell line**	PC12 TetOff for MK5L337A			C2C12			SK-N-DZ		HeLa	
**Requested Comparisons**	1. Normal vs MK5 Induced			2. siRNA#1 vs Scrambled 3. siRNA#2 vs Scrambled			4. siRNA vs Scrambled		5. siRNA vs Scrambled	
**Blind analysis**	Microarray facilityTromsø Loess normalization			UNIGEN (Trondheim) Quantile Normalization			UNIGEN (Trondheim) Quantile Normalization			
	27468 reported of which 4007 in agreement			0	0		not submitted		70	
**Intensity dependent analysis**	Both Quantile and no normalization			Applied Biosystems Inter-array normalization			Applied Biosystems Inter-array normalization			
	1422			2977	576		661		2497 (22 validated through qPCR)	
**Overlap**	311, with 10 wrong in the standard analysis			0	0		not applicable		65	

**Table 2:** Gene regulation induced by MK5 activation. Each regulation is listed as a term with a confidence interval covering 95% of the real values. Gene regulation is calculated as the mean of all the measured oligosequences/probes. The reported confidence interval is the result of a convolution of the respective error distributions. The yellow row is explained in detail in the text.

		Difference			Summed Values			Regulation Ratio			
		
Gene #	Confidence Interval	At least	Measured	At most	Green	Red	Count	Direction	At least	Measured	At most
1	[−6430.72:6840.32]	−39267.7	−32837	−25996.7	39613	6776	2	down	4.84	5.85	114.73
2	[−2447.36:2242.56]	−7807.36	−5360	−3117.44	6191	831	2	down	4.75	7.45	inf
3	[−2355.2:2129.92]	−5122.2	−2767	−637.08	3807	1040	2	down	1.61	3.66	inf
4	[−2775.04:2754.56]	1531.96	4307	7061.56	2531	6838	6	up	1.61	2.7	inf
5	[−2437.12:2447.36]	−5919.12	−3482	−1034.64	5215	1733	4	down	1.6	3.01	inf
6	[−2037.76:2457.6]	472.24	2510	4967.6	809	3319	2	up	1.58	4.1	inf
7	[−3532.8:3430.4]	2701.2	6234	9664.4	4818	11052	4	up	1.56	2.29	inf
8	[−1812.48:1536]	−3697.48	−1885	−349	2514	629	2	down	1.55	4	inf
9	[−2590.72:2621.44]	−6302.72	−3712	−1090.56	5684	1972	6	down	1.55	2.88	inf
10	[−2170.88:2314.24]	969.12	3140	5454.24	1854	4994	6	up	1.52	2.69	inf
11	[−3461.12:3686.4]	2038.88	5500	9186.4	3982	9482	2	up	1.51	2.38	32.08
12	[−2283.52:2048]	−5168.52	−2885	−837	4528	1643	2	down	1.51	2.76	inf
13	[−8448:8704]	10789	19237	27941	21540	40777	2	up	1.5	1.89	3.18
14	[−2754.56:3368.96]	765.44	3520	6888.96	1555	5075	2	up	1.49	3.26	inf
15	[−1771.52:1986.56]	438.48	2210	4196.56	914	3124	2	up	1.48	3.42	inf
16	[−6082.56:5898.24]	5740.44	11823	17721.2	12046	23869	2	up	1.48	1.98	3.88
17	[−2078.72:2211.84]	−4762.72	−2684	−472.16	3708	1024	2	down	1.46	3.62	inf
18	[−2119.68:2037.76]	−4787.68	−2668	−630.24	4044	1376	2	down	1.46	2.94	inf
19	[−1781.76:1792]	314.24	2096	3888	688	2784	2	up	1.46	4.05	inf
20	[−3932.16:4259.84]	2675.84	6608	10867.8	5984	12592	2	up	1.45	2.1	7.3
21	[−10455:10915.8]	−36683	−26228	−15312.2	85832	59604	2	down	1.26	1.44	1.75
22	[−7700.48:7782.4]	5041.52	12742	20524.4	20556	33298	2	up	1.25	1.62	2.61
23	[−2140.16:2273.28]	320.84	2461	4734.28	1321	3782	2	up	1.24	2.86	inf
24	[−2621.44:2979.84]	−6161.44	−3540	−560.16	5883	2343	2	down	1.24	2.51	inf
25	[−3450.88:3952.64]	−8665.88	−5215	−1262.36	10529	5314	2	down	1.24	1.98	5.65
26	[−2232.32:2600.96]	−5150.32	−2918	−317.04	4264	1346	4	down	1.24	3.17	inf
27	[−2181.12:2099.2]	202.88	2384	4483.2	867	3251	2	up	1.23	3.75	inf
28	[−3758.08:3768.32]	1212.92	4971	8739.32	5296	10267	4	up	1.23	1.94	6.72
29	[−4925.44:5857.28]	2682.56	7608	13465.3	11941	19549	2	up	1.22	1.64	3.21
30	[−2426.88:2887.68]	418.12	2845	5732.68	1909	4754	2	up	1.22	2.49	inf
31	[−5980.16:5867.52]	−14564.2	−8584	−2716.48	20997	12413	2	down	1.22	1.69	3.26
32	[−4423.68:4966.4]	−11228.7	−6805	−1838.6	15221	8416	4	down	1.22	1.81	3.81
33	[−1771.52:1484.8]	−3399.52	−1628	−143.2	2307	679	2	down	1.21	3.4	inf
34	[−3491.84:3481.6]	331.16	3823	7304.6	5513	9336	4	up	1.06	1.69	4.6

**Table 3:** Wrongly reported datapoints in the loess normalized data. We compared the regulations of our method to a standard loess normalization and found 10 spots for which the two methods disagreed qualitatively. Each case contains the data as found on the non-normalized microarray (reported in the two first green/red columns). The reported log ratio after loess normalization is given in the second row of each case. The reported confidence interval is presented in the first row of each case.

confidence intervals	C.I.	Difference			Values			Regulation	Factor		
		Low	Norm	Hi	Green	Red	Count		Lo	Mes	
		
loess					D4D1	D6D3					
		
non-normalized	Slide 1		Slide 3								
	Green	Red	Green	Red							
Rn 30026543	[− 3983.36:3993.6]	−277.64	−4261	−8254.6	10661	6400	2	down	1.03	0.6	confidence intervals
					0.49	0.55		up			loess
	4336	2218	7262	3661							non-normalized

Rn 30009746	[−1904.64:1812.48]	−41.36	−1946	−3758.48	2743	797	2	down	1.02	0.29	confidence intervals
					0.12	0.02		up			loess
	911	683	2001	113							non-normalized

Rn 30025831	[−2918.4:3246.08]	−545.6	−3464	−6710.08	8138	4674	2	down	1.09	0.57	confidence intervals
					0.21	0.41		up			loess
	2274	1383	6508	2910							non-normalized

Rn 30026511	[−8212.48:8407.04]	−1460.52	−9673	−18080	41854	32181	2	down	1.04	0.77	confidence intervals
					0.43	0.06		up			loess
	10631	8385	34489	20727							non-normalized

Rn 30023124	[−5539.84:5683.2]	11256.8	5717	33.8	14262	19979	2	up	1	1.4	confidence intervals
					−0.13	−0.11		down			loess
	7556	8168	8065	10364							non-normalized

Rn 30026938	[−2959.36:2826.24]	−580.64	−3540	−6366.24	5297	1757	2	down	1.18	0.33	confidence intervals
					0.02	0.02		up			loess
	2104	827	3590	880							non-normalized

Rn 30026618	[−7618.56:8785.92]	17364.6	9746	960.08	109415	119161	2	up	1.01	1.09	confidence intervals
					−0.01	−0.13		down			loess
	53944	57496	57493	60704							non-normalized

Rn 30026891	[−2939.88:3481.6]	−444.12	−3383	−6864.6	7455	4072	2	down	1.08	0.55	confidence intervals
					0.01	0.1		up			loess
	2347	1004	5737	2757							non-normalized

Rn 30000378	[−6338.56:7075.84]	13860.6	7522	446.16	114279	121801	2	up	1	1.07	confidence intervals
					−0.26	−0.12		down			loess
	63294	64294	53346	56126							non-normalized

Rn 30018614	[−1904.64:1853.44]	3883.64	1979	125.56	851	2830	2	up	1.07	3.33	confidence intervals
					−0.1	0		down			loess
	528	958	311	1711							non-normalized

**Table 4:** Quantitative PCR analysis to verify differentially expressed genes. A number of the genes that were reported to be expressed differentially by the microarray analysis were measured using quantitative PCR.

				qPCR results		Microarray results			

TAF4	#	Mean	CT	Ratio	Fixed *1	Ratio	least	most	Comments
Hela Cells	1	29.88	down	1.33	1.6	down	1.2	2.45	OK
	2	29.72	down	1.32	1.59	up	1.07	1.66	NO, *2
	3	29.41	up	1.03	1.24	up	1.22	1.78	OK
	4	30.84	up	1.09	1.32	up	7.84	inf	NO, *6
	5	25.46	up	2.76	3.34	up	2.64	5.01	OK, *6
	6		down	large	large	down	122.53	inf	OK, *3,6
	7	38.93	down	2.67	3.23	down	3.57	inf	OK, *3
	8	38.26	down	1.25	1.52	down	3.18	8.5	OK, *3
	9	34.02	up	1.04	1.26	up	1.13	1.88	OK
	10	31.1	down	1.2	1.45	down	1.22	2	OK
	11	26.09	down	1.02	1.23	down	1.23	1.91	OK
	12	35.48	up	1.38	1.67	up	1.03	1.59	NO, *4
	13	34.03	down	1.05	1.27	up	1.1	1.65	NO, *2,5
	14	35.99	up	1.03	1.25	down	1.11	1.54	NO, *2,5
	15	31.38	down	2.04	2.47	down	1.5	2.23	NO, *4
	16	31.01	up	1.06	1.28	up	1.08	1.65	OK
	17	34.67	up	1.49	1.8	up	1.36	3.32	OK

SK-N-DZ Cells	18	28.73	down	1.47	1.47	down	1.16	1.7	OK
	19	28.15	down	1.52	1.52	down	1.03	1.98	OK
	20	35.02	up	1.38	1.38	up	1.06	2.96	OK
	21	33.11	down	1.49	1.49	down	1.09	1.87	OK
	22	38.04	up	1.24	1.24	down	14.99	inf	NO, *2,3

All results are reported as a ratio from the scrambled siRNA to the specific siRNA

* 1) HeLa cells results have been multiplied to account for transfection efficiency; 2) Regulation direction reported wrong; 3) qPCR result difficult to obtain due to large CP values; 4) Microarray upperbound too low; 5) Difficult consensus on PCR results; 6) Also listed in the genvar analysis

## Footnotes

1 SiRNA will bind to the transcript and activate the destruction or prevent translation of the target sequence (Elbashir, 2001).

2 The TAF4 SK-N-DZ was not sent for analysis, but to be complete, we found 661 to be significant.
